# Diatoms: A Novel Source for the Neurotoxin BMAA in Aquatic Environments

**DOI:** 10.1371/journal.pone.0084578

**Published:** 2014-01-02

**Authors:** Liying Jiang, Johan Eriksson, Sandra Lage, Sara Jonasson, Shiva Shams, Martin Mehine, Leopold L. Ilag, Ulla Rasmussen

**Affiliations:** 1 Department of Ecology, Environment and Plant Sciences, Stockholm University, Stockholm, Sweden; 2 Department of Analytical Chemistry, Stockholm University, Stockholm, Sweden; 3 Sustainable Agro-ecosystems and Bioresources Department, IASMA Research and Innovation Centre - Fondazione Edmund Mach, Trento, Italy; Stazione Zoologica, Italy

## Abstract

Amyotrophic lateral sclerosis (ALS) or Lou Gehrig’s disease is a neurological disorder linked to environmental exposure to a non-protein amino acid, β-*N*-methylamino-L-alanine (BMAA). The only organisms reported to be BMAA-producing, are cyanobacteria – prokaryotic organisms. In this study, we demonstrate that diatoms – eukaryotic organisms – also produce BMAA. Ultra-high-performance liquid chromatography coupled with tandem mass spectrometry revealed the occurrence of BMAA in six investigated axenic diatom cultures. BMAA was also detected in planktonic field samples collected on the Swedish west coast that display an overrepresentation of diatoms relative to cyanobacteria. Given the ubiquity of diatoms in aquatic environments and their central role as primary producers and the main food items of zooplankton, the use of filter and suspension feeders as livestock fodder dramatically increases the risk of human exposure to BMAA-contaminated food.

## Introduction

β-*N*-methylamino-L-alanine (BMAA) is a neurotoxic non-protein amino acid found by many studies to be strongly linked to the neurodegenerative disease amyotrophic lateral sclerosis (ALS) [1]–[6], also known as Lou Gehrig’s disease. BMAA was first discovered in 1967, when it was isolated from the seeds of *Cycas circinalis* and was supposed to be a plant-derived toxin [7]. It was later proven to be synthesized by cyanobacteria (*Nostoc* sp.), living symbiotically in the coralloid roots of cycads and to accumulate in their seeds [4]. Human exposure to BMAA and thus potential impact on human health is caused by the bioaccumulation and transfer of BMAA to higher trophic levels. This has been reported in terrestrial as well as in aquatic ecosystems [4], [8].

The first instance of BMAA accumulation and human exposure [5], [9], [10] was recorded on the island of Guam in the Western Pacific Ocean where it was demonstrated that cyanobacteria in the coralloid roots of a Cycad (*Cycas micronesica*) produced BMAA. BMAA was then transferred into the seeds of the cycad; fruit bats ate the seeds, bioaccumulating the BMAA, which was transferred to Guam’s indigenous people, the Chamorro, through their consumption of the fruit bats [2], [5], [9], [11]. Brain autopsy material from the Chamorro displaying ALS pathology all contained BMAA, which strengthened the theory of BMAA involvement in ALS [6].

We have previously demonstrated that BMAA produced in a temperate aquatic ecosystem, i.e., the Baltic Sea, can be transferred from cyanobacteria to zooplankton and accumulate in various vertebrates and invertebrates leading to potential human exposure [8]. Bioaccumulation of the toxin has also been found in a subtropical ecosystem in Florida Bay [12], [13]. In one of these studies, high BMAA content was primarily found in filter-feeding organisms (e.g., oysters and mussels) and organisms at lower trophic levels such as the blue crab (*Callinectes sapidus*). In the Florida Bay area, massive blooms of phytoplankton including cyanobacteria, diatoms, dinoflagellates, and flagellates frequently occur [14]. These findings, taken together with the presence of BMAA in mussels collected on the Swedish west coast, a marine environment dominated by blooms of diatoms and dinoflagellates [15], prompted the present investigation of whether or not diatoms or other phytoplankton might be an additional source of BMAA production.

Diatoms are the most frequent group of algae, representing 40% of the primary production in the Ocean today [16]–[18], making them the globally most abundant photosynthetic group after angiosperms. Because of their abundance in the marine phytoplankton community, especially in nutrient-rich areas of the world’s oceans, diatoms probably account for as much as 20% of global photosynthetic fixation of carbon [19]; as such, diatoms occupy a central position in the control of marine resources. Based on species composition, diatoms are also a commonly used tool for determining freshwater quality [20].

Some diatoms species are known to produce domoic acid (amnesic shellfish toxin) and other non-toxic compounds, which are harmful for fish and invertebrates (damaging or clogging their gills) [21]. Despite this, there are several documented cases of acute intoxication of wildlife from diatom blooms in various parts of the world. Due to their inherent morphology, diatoms have a propensity to become less buoyant and to settle with the sediment, providing benthic pathways for the contamination of soft-bottom-dwelling vertebrates and invertebrates [20], [22], [23]. Benthic pathways have previously been shown to be the major route for BMAA accumulation e.g. mussels, crabs, oysters, shrimps as well as bottom dwelling and feeding fish such as turbot, sculpin and parrot fish [8], [12]. Due to the low buoyancy of diatoms they tend to sink to the bottom when blooms degenerate and therefore potentially expose the benthic fauna to high levels of BMAA. This provides routes for toxin transfer to top predators, such as cephalopods [24], and to other secondary consumers, as well as potentially poisoning humans consuming contaminated shellfish [25], [26]. Therefore we hypothesized that phytoplankton organisms could be a new potential source for BMAA in aquatic environment and thus increasing our understanding of possible human exposure routes to this toxin.

## Materials and Methods

### Sample Collection and Culture Conditions

On the present work, no specific permissions were required to collect samples of planktonic and benthic cyanobacteria from the Swedish west coast; and no protected species were collected.

Axenic diatom cultures of *Navicula pelliculosa* (CCAP 1050/9), *Thalassiosira* sp. (CCAP 1085/15), *Achnanthes* sp. (CCAP 1095/1), and *Proboscia inermis* (CCAP 1064/1) were obtained from the Culture Collection of Algae and Protozoa, Scottish Marine Institute, Dunbeg, Oban, Scotland, and the two *Skeletonema marinoi* isolates (SAAE 08603 and ST 28) were kindly provided by Prof. Anna Godhe, Department of Biological and Environmental Sciences, University of Gothenburg, Sweden. All cultures were grown in f/2+Si medium [27] at 15°C with slight agitation and under a 12-h light, 12-h dark cycle at a light intensity of 40–50 µmol s^–1^ m^–1^.

Pelagic and benthic field samples were collected near Kristineberg Marine Research Station, at the mouth of the Gullmar Fjord, on the Swedish west coast (58.2°N, 11.3°E) in 2010. The cultures were grown in SO medium [28] at 15°C under the same conditions as above. The cultures were subjected to several rounds of plating on SO agar plates to enrich individual cyanobacterial morphotypes. The dominant cyanobacterial and diatom morphotypes in the individual samples were determined by microscopy analysis using an Olympus BH-2 microscope equipped with a digital camera.

The field sample of *Leptolyngbya* sp./*Naviculales* was treated with germanium dioxide for three weeks in a final concentration of 10 mg L^–1^ in order to eliminate the diatoms, according to the method of Andersen [29]. The culture was grown under same conditions as above and regularly observed using light microscopy.

### Sample Preparation

All samples were centrifuged at 4100×g for 15 min. The supernatant was discarded and the pellet was stored at –20°C while awaiting analysis. The pooled samples were dissolved in 20% methanol in water (v/v), frozen at –80°C, and then lyophilized to determine the dry weight. The lyophilized material was then dissolved in 2 mL of 20% methanol in water (v/v) and sonicated for 40 min at 70% efficiency (Sonopuls, Model HD 2070, Bandelin Electronic, Berlin, Germany); the material was kept cool in an ice/methanol bath to prevent protein degradation.

The cell solution was centrifuged at 4100×g for 20 min at 4°C, and the resulting pellet was stored at –20°C. The supernatant was transferred into new tubes and twice the supernatant volume of 4°C acetone was added. The sample was allowed to precipitate at –20°C overnight. The solution was centrifuged at 4100×g for 20 min at 4°C and the supernatant was discarded. The resulting pellet containing the protein fraction was lyophilized to determine the dry weight. To hydrolyze the proteins into amino acids, 600 µL of 6 M HCl was added to the protein pellet and the sample was hydrolyzed overnight (22 h) at 110°C. The hydrolysate was then filtered using Ultrafree spin filters (Millipore, Billerica, MA, USA) to remove particulate materials and frozen at –80°C before lyophilization using a miVac centrifugal vacuum concentrator (Genevac, Ipswich, UK) connected to a freeze-dryer (CoolSafe, SCANVAC). Finally, the dried hydrolysate was reconstituted in 20 µL of 20 mM HCl solution before derivatization with AccQ-Tag using a WAT052880 AccQ-Tag kit (Waters, Milford, MA, USA). Broccoli (*Brassica oleracea*) and cycad seed (*Cycas Revoluta)* were used as negative and positive controls, respectively, and were subjected to the entire sample pretreatment process. An AccQ-Tag reagent blank was injected between successive samples to determine whether there was any carry-over in the system.

To quantify BMAA in the protein pellet of the diatom samples, 10 µL of deuterated BMAA internal standard (250 ng mL^–1^) was added to the protein pellet in triplicate before acid hydrolysis to compensate for the loss of BMAA during subsequent sample preparation. The samples were then treated as described above.

### UHPLC-MS/MS Analysis

**Figure 1:**
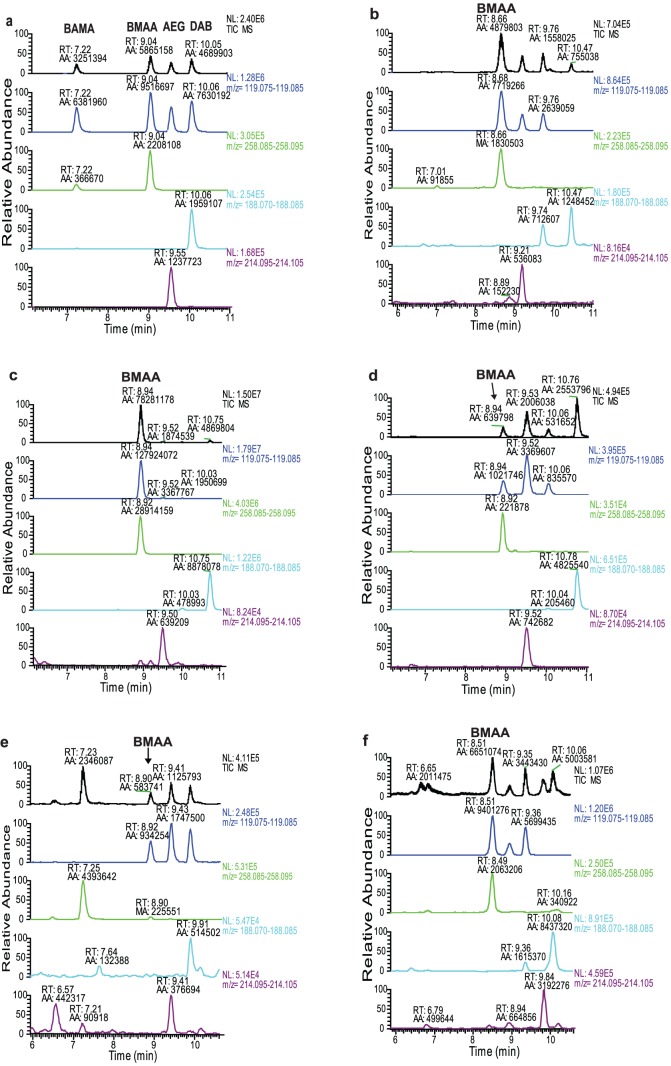
LC-MS/MS chromatograms of (a) BMAA and its isomer standards (5 µg L^–1^ for BAMA, BMAA, and AEG and 20 µg L^–1^ for DAB), and chromatograms showing BMAA produced by axenic cultures of diatoms; (b) *Achnanthes* sp. CCAP 1095/1; (c) *Navicula pelliculosa* CCAP 1050/9; (d) *Skeletonema marinoi* SAAAE 08603; (e) *Skeletonema marinoi* ST28; (f) *Thalassiosira* sp. CCAP 1085/15; (g) *Proboscia inermis* CCAP 1064.

UHPLC was performed using an AccQ-Tag Ultra C18 column (100×2.1 mm, 1.7 µm particle size, Waters, Ireland) with an Accela pump and Accela autosampler (Thermo Fisher Scientific, San Jose, CA, USA). Mass spectrometry was carried out on a TSQ Vantage triple quadrupole mass spectrometer (Thermo Fisher Scientific). Xcalibur 2.1 software was used to analyze the acquired data. Settings were as reported by Jiang et al. [30]. The BMAA was identified using a highly selective LC-MS/MS method which unambiguously distinguishes BMAA from its isomers, such as BAMA, AEG, and DAB, in biological samples (Fig. 1a). The BMAA in diatoms was quantified using a six-point calibration curve constructed with a dilution series of BMAA standards (0.2–10 ng) and a fixed amount of deuterated BMAA as an internal standard (2.5 ng) in 2 mg of cyanobacterial matrix [31]. The samples were analyzed in triplicate.

## Results

### BMAA in Axenic Diatom Cultures and Field Samples

BMAA was detected in the six axenic diatom cultures investigated in this study (Table 1) and in field samples collected on the Swedish west coast (Table 2).

**Table 1:** BMAA in axenic diatom cultures.

Species	Strain number	Collection place	Habitat	BMAA
*Achnanthes* sp.	1CCAP 1095/1	Millport, Scotland	Marine	D (*n* = 2)
*Navicula pelliculosa*	1CCAP 1050/9	Massachusetts, USA	Marine	D (*n* = 3)
*Proboscia inermis*	1CCAP 1064/1	Brandsfield Strait, 63°15′S 58°20′W	Marine	D (*n* = 3)
*Skeletonema marinoi*	SAAE08603	Gullmarsfjorden, Sweden	Marine	D (*n* = 1)
*Skeletonema marinoi*	ST28	Strömstad, Sweden	Marine	D (*n* = 3)
*Thalassiosira* sp.	1CCAP 1085/15	Loch Linnhe, UK 56°28′N 50°30′W	Marine	D (*n* = 3)

^1^ Culture Collection of Algae and Protozoa.

D: Detected.

*n*: Biological replicates.

**Table 2:** BMAA in field samples collected on the Swedish west coast.

Dominant cyanobacteria/diatom species or order	Collection place	Habitat	BMAA
*Leptolyngbya* sp./*Naviculales*	Kristineberg	Marine (planktonic)	D
*Calothrix* sp.	Kristineberg	Marine(benthic)	ND
*Rivularia* sp.	Kristineberg	Marine(benthic)	ND
*Oscillatoria* sp./*Nitzshia* sp.	Kristineberg	Marine (planktonic)	ND*

D: detected; ND: not detected.

The peak had too low intensity to be determined as a positive sample.

The benthic and planktonic field samples collected near Kristineberg Marine Research Station on the Swedish west coast were categorized by microscopy analysis according to dominant cyanobacterial and diatom morphotype (Table 2). The results of ultra-high performance liquid chromatography coupled with tandem mass spectrometry (UHPLC-MS/MS) analysis indicated a link between the presence of BMAA and the abundance of diatoms in the cultures. For example, in the planktonic field samples dominated by the cyanobacteria *Leptolyngbya* sp. and diatoms from the order *Naviculales*, a peak corresponding to BMAA was seen with high intensity in the LC-MS/MS chromatogram. In contrast, the sample dominated by *Oscillatoria* sp., but with a low abundance of the diatom *Nitzshia* sp., the chromatographic peak corresponding to BMAA had too low intensity to confidentially determine it as a positive sample (Table 2). In the two samples dominated by the benthic cyanobacteria *Rivularia* sp. or *Calothrix* sp., no diatoms could be detected by microscopy analysis nor could any BMAA be detected (Table 2). This could indicate that diatoms might be the BMAA producer in these mixed field samples. To investigate this hypothesis, five axenic species of diatoms (Table 1) were cultured and analyzed; BMAA was detected in all of the cultures (Fig. 1b–g). No BMAA was detected in either broccoli (*Brassica oleracea*) or 6-aminoquinolyl-*N*-hydroxysuccinimidyl carbamate (AccQ-Tag) reagent samples used as negative controls. The content of protein-bound BMAA varied between 1.1 ng/g dry weight to 3.8 ng/g dry weight in three investigated discrete samples (Table 3). The sample workup was designed to detect protein-bound or protein-associated amino acids. The level of free BMAA was not analyzed, as we were focusing on BMAA associated with/incorporated into the protein fraction. A modification of our previously described sample workup [32] was undertaken as unknown substances from the diatoms strongly interfered with the chromatographic separation and caused column failure (see Materials and Methods).

**Table 3:** Quantification of BMAA in diatoms.

Species	BMAA/dry weight (ng/g)*
*Skeletonema marinoi* SAAE08603	1,1±0,4 (*n* = 2)
*Skeletonema marinoi* ST28	1,07±0,8 (*n* = 2)
*Thalassiosira* sp. CCAP 1085/15	3,28±2,5 (*n* = 3)

Mean of BMAA concentration (±STD) in different samples of diatoms.

*n*: Biological replicates.

**Figure 2:**
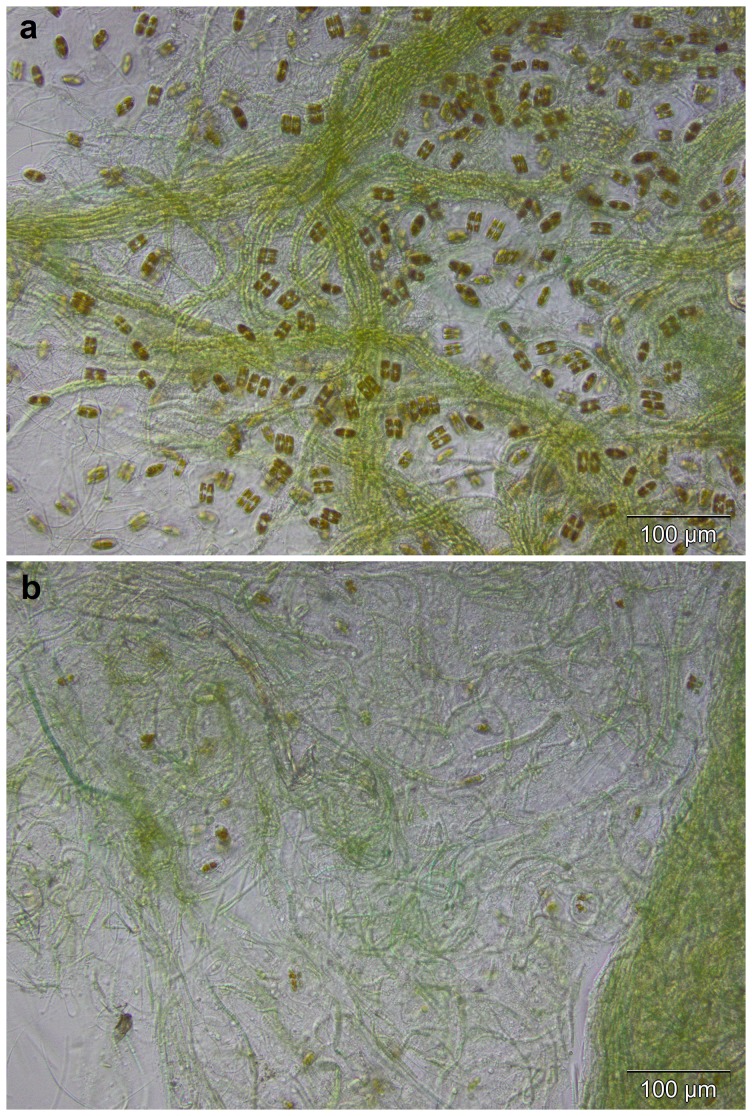
Microphotographs of the field culture *Leptolyngbya* sp./Naviculales. (**a**) untreated culture; (**b**) germanium dioxide-treated culture.

Furthermore, to investigate whether or not the BMAA peak detected in the diatom-containing field samples was associated with the presence of diatoms, an experiment was conducted in which the mixed *Leptolyngbya* sp./*Naviculales* culture was treated with germanium dioxide. After three weeks of treatment, microscopy analysis of the culture revealed a highly reduced amount of diatom presence (Fig. 2). Analysis of the treated sample gave an approximate three times reduction in concentration of BMAA compared to the untreated sample (Table 4), suggesting that the amount of BMAA in the sample was to a great extent contributed by the diatoms.

**Table 4:** Germanium dioxide treated and non-treated field samples.

Treatment	BMAA/dry weight(ng/g)*
Germanium dioxide	4,7±1,7 (*n* = 3)
Non treated	27,6±11 (*n* = 2)
Negative control (*Brassica oleracea*)	Negative
Positive control (*Cycas Revoluta*)	Positive

Mean of BMAA concentration (±STD) in different samples of diatoms.

*n*: Biological replicates.

## Discussion

This is the first report of the production of BMAA outside the cyanobacteria phylum and the first showing that diatoms – eukaryotic organisms – can produce BMAA. Since 2003 when BMAA production by cyanobacteria was first reported [4], [33], researchers have speculated whether they were the only organisms with this capacity and, if so, why.

Controversial results reported over the last 10 years of BMAA production by cultured cyanobacteria, obtained using a variety of analytical approaches and sample pre-treatments, have created confusion in the research field [32]–[38]. In this study, a novel sample workup strategy suitable for the detection of protein-bound BMAA in diatom samples was developed. By using cold acetone to precipitate proteins, a clean protein pellet was obtained and unknown substances from the diatoms which seriously damaged the LC column were removed. The LC-MS/MS method used here can distinguish BMAA from its three isomers, i.e. β-amino-*N*-methyl-alanine (BAMA), 2, 4-diaminobutyric acid (DAB) and *N*-(2-aminoethyl) glycine (AEG) using chromatographic retention time, diagnostic fragments and fragments ratio, leading to highly reliable identification of BMAA [30], [31]. The AEG and DAB isomers are detected in some of the axenic diatom cultures (Fig. 1b–g), although the biological significance of these BMAA isomers is unclear. AEG has been suggested to be a building block of peptide nucleic acid (PNA) which is a molecule believed to precede ribonucleic acid (RNA) [39].

Our five axenic diatom species were selected based on their habitual occurrence in the Baltic Sea. BMAA was detectable in all tested samples, suggesting that BMAA production might be common among diatoms. Due to their morphology, diatoms can become attached to various substrates, such as plants, rocks, glass slides, and sides of ships – any natural or artificial submerged substrate, including bird legs and feathers as well as the scales of fish – which accounts for their worldwide spread [21], [40], [41]. As diatoms are as ubiquitous as cyanobacteria in aquatic systems and are important primary producers, diatoms play a key role in marine food webs and provide an important food item for both zooplankton and filter and suspension feeders, such as worms, anemones, and mollusks, which are subsequently consumed by secondary consumers and top predators. This is of utmost importance concerning the health risks to humans, since BMAA has been shown to bioaccumulate in the food web [6], [8], [11]–[13]. In addition, as mentioned earlier, diatoms and cyanobacteria tend to descend in the water column and decompose in the bottom sediments. This could also explain earlier findings of an increase in BMAA bioaccumulation in bottom-dwelling/feeding organisms.

Our finding of BMAA in both diatom-containing field samples and the six axenic diatom cultures suggests that diatoms might have a substantial impact on the overall abundance of BMAA in aquatic environments. Diatoms are the most diverse group of phytoplankton, with an estimated 200,000 species [42], and tend to dominate phytoplankton communities in well-mixed coastal and upwelling regions, where light, inorganic nitrogen, phosphorus, silicon, and trace elements are available in sufficient amounts to sustain their growth [43]. The data on the field samples indicate no clear relationship between BMAA and benthic cyanobacteria, which disproves a hypothesis that benthic cyanobacteria may produce higher levels of BMAA than do planktonic cyanobacteria. This hypothesis could have explained the high BMAA content in mussels and bottom-dwelling/feeding organisms. In addition, our axenic diatom cultures consistently expressed BMAA over time, unlike cyanobacteria in culture which display fluctuating BMAA production. The levels of bound and free BMAA detected in cultured cyanobacteria have been suggested to be in the low nanograms per milligram or picograms per milligram dry weight [35]. In a recent publication, the level of bound and free BMAA produced by a cyanobacteria (*Leptolyngbya* PCC 73110) was reported to be 0.73±7%µg/g (*n* = 3) dry weight [31]. An isotopic-labeled internal standard quantification strategy was used to quantify the BMAA content in three diatom cultures (Table 3). The average BMAA concentration in the diatom cultures is lower based on dry weight compared to cyanobacteria. However, cyanobacteria and diatoms can not be compared directly on a dry weight basis, as one mg of diatoms cells contains less protein than one mg of cyanobacteria cells, due to the weight of the diatom frustule. In addition, it is worth keeping in mind the sample workup strategy used in this study where we only measured the associated/protein incorporated BMAA in contrast to the method used for cyanobacteria where both protein bound and free BMAA were analyzed. Finally, our results from the germanium dioxide-treated field samples in which less BMAA were detected (compared with untreated samples) strengthen our finding that BMAA is not produced only by axenic cultured diatoms but also in field samples. Germanium dioxide is commonly used to inhibit diatom growth in mixed samples with cyanobacteria, as germanium dioxide, instead of silica, is incorporated into the diatom frustule, but leaves cyanobacteria unaffected [29]. It is noteworthy that the measurement is normalized against the dry weight of the samples. The untreated sample is a mixture of diatom and cyanobacterial cells whereas the treated is dominated by cyanobacterial cells. Thus, the contribution of BMAA from the diatoms might be percentage larger than here presented, as we do not know the cyanobacteria:diatom ratio in the mixed sample. This is also important when sampling cyanobacteria for BMAA analysis, as diatom presence must be monitored in the samples to rule out BMAA contamination from diatoms. It is noteworthy that axenic culture of dinoflagellates also contained BMAA when analyzed, but this need further investigation to be conclusive.

Taken together, the data reported here give a clear answer supported by solid evidence that BMAA is not exclusively produced by cyanobacteria. As diatoms are a major bloom-forming phytoplankton in aquatic environments, the impact of this discovery suggests new bioaccumulation routes and that the risk of human exposure may have increased tremendously. This stresses the need for a modeling and monitoring approach to the ecological spread of BMAA and a concern for the potential socio–economic impact of BMAA accumulation in organisms with high commercial value. If these eukaryotic organisms can produce BMAA, it is highly likely that other eukaryotic organisms have the capacity as well. Our findings also help resolve the question of why BMAA is continuously being reported in field samples, versus in axenic cultured cyanobacteria. This highlights the crucial importance of knowing the community structure of a field sample in order to draw conclusions as to the origin of BMAA.
