# Correction: Diatoms: a novel source for the neurotoxin BMAA in aquatic environments

**DOI:** 10.1371/journal.pone.0106695

**Published:** 2014-08-20

**Authors:** The PLOS ONE Staff

Figure 1(G) is missing from the original publication. A complete Figure 1, provided by the authors, can be viewed here.

**Figure 1:**
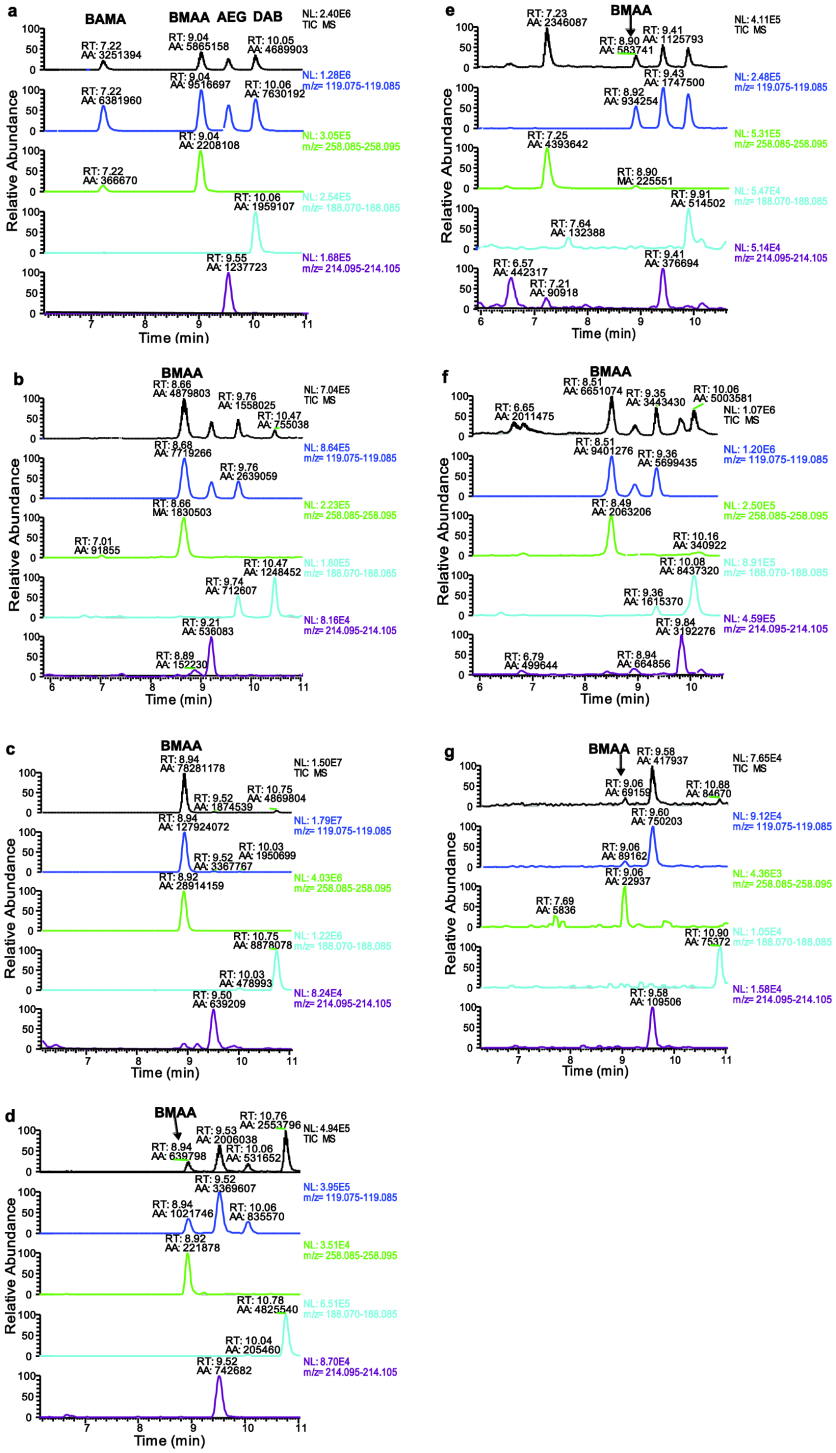
LC-MS/MS chromatograms of (a) BMAA and its isomer standards (5 µg L^–1^for BAMA, BMAA, and AEG and 20 µg L^–1^ for DAB), and chromatograms showing BMAA produced by axenic cultures of diatoms; (b) *Achnanthes* sp. CCAP 1095/1; (c)*Navicula pelliculosa* CCAP 1050/9; (d) *Skeletonema marinoi* SAAAE 08603; (e)*Skeletonema marinoi* ST28; (f) *Thalassiosira* sp. CCAP 1085/15; (g) *Proboscia inermis* CCAP 1064.
